# A comprehensive analysis reveals the relationship between artificial sweeteners and prostate cancer

**DOI:** 10.3389/fnut.2025.1646623

**Published:** 2025-09-10

**Authors:** Kuiyuan Zhang, Bangwei Che, Pudong Gao, Wei Li

**Affiliations:** ^1^Department of Urology, First Affiliated Hospital of Guizhou University of Traditional Chinese Medicine, Guiyang, China; ^2^Department of Urology, Guizhou Provincial People's Hospital, Guiyang, China; ^3^Department of Urology, Affiliated Hospital of Guizhou Medical University, Guiyang, China

**Keywords:** artificial sweetener, prostate cancer, robot learning, molecular docking, matrix metalloprotein 11

## Abstract

**Background:**

Global consumption of artificial sweeteners (ASs) has risen substantially in recent years. However, their relationship with prostate cancer (PCa) remains poorly characterized. This study investigates the AS–PCa association to identify pivotal genes potentially bridging this relationship.

**Method:**

This study retrieved target genes associated with ASs and PCa from multiple public databases. Protein–protein interaction (PPI) network analysis and visualization were conducted on overlapping genes, followed by the Gene Ontology (GO) and Kyoto Encyclopedia of Genes and Genomes (KEGG) enrichment analyses to explore the underlying mechanisms. Subsequently, the optimal predictive model was selected from 101 machine-learning algorithm combinations and validated against 2 external datasets. Molecular docking analysis was then performed to examine the interactions between key genes and AS compounds. Finally, *in vitro* cellular assays were conducted to validate the specific effects of ASs on PCa.

**Results:**

We analyzed seven common ASs—aspartame, acesulfame-K, sucralose, NHDC, sodium cyclamate, neotame, and saccharin—identifying 261 overlapping targets associated with PCa. The GO and KEGG enrichment analyses revealed that these targets primarily regulate cell proliferation, inflammation, and cancer cell metabolism. Machine learning algorithm screening identified the Lasso-SuperPC hybrid model as demonstrating optimal predictive performance, with robust validation in two independent external datasets. Subsequent analysis identified two key regulatory genes: CD38 and MMP11. Molecular docking analysis further confirmed potential interactions between AS compounds and the core target MMP11. Finally, *in vitro* cellular assays demonstrated that NHDC suppresses MMP11 expression in PCa cells and exhibits anti-PCa pharmacological effects.

**Conclusion:**

By integrating bioinformatics, machine learning, molecular docking, and *in vitro* cellular assays, this study demonstrates that ASs inhibit PCa progression through multiple molecular targets and signaling pathways. Collectively, these findings provide important insights into the safety assessment of food additives and cancer risk assessment.

## Background

Artificial sweeteners (ASs) are low- or zero-calorie sugar substitutes extensively used in the food, beverage, and pharmaceutical industries, with globally rising consumption (1, 2). Despite their low-calorie properties (200–13,000 times sweeter than sucrose), which makes them suitable for diabetics and individuals on sugar-restricted diets (2–4), the long-term safety of AS consumption remains inconclusive. The Joint FAO/WHO Expert Committee on Food Additives (JECFA), the authoritative evaluative body, establishes acceptable daily intakes (ADIs) through comprehensive assessments including acute toxicity and carcinogenicity studies. JECFA maintains that ADI-compliant usage poses no health risks (4). Furthermore, as most ASs are synthesized from natural precursors, they exhibit pharmacological properties including antipyretic, analgesic, anti-inflammatory, and immunomodulatory effects (5–7). However, despite three decades of dietary use, significant research interest persists regarding their toxicological profiles, particularly their carcinogenic potential. Several studies suggest potential cancer risks associated with AS intake (4, 8–10). Yet, inconsistent epidemiological evidence in humans leaves the carcinogenic significance of AS controversial.

Prostate cancer (PCa) represents the most prevalent malignancy and the second-leading cause of cancer-related mortality among men worldwide (11, 12). With approximately 1.4 million new cases and 396,000 deaths projected for 2024, PCa poses a major health threat and substantial healthcare burden (11, 12). Established PCa risk factors include age, ethnicity, and family history, as highlighted in prior research. Nevertheless, the influence of environmental determinants, lifestyle factors, and dietary components on PCa pathogenesis remains incompletely understood (13, 14). Although numerous studies have examined AS consumption–PCa risk associations, conclusive evidence remains elusive (4, 9, 15). Paradoxically, some ASs demonstrate antitumor properties potentially beneficial for PCa management, partly attributable to their antipyretic, analgesic, anti-inflammatory, and immunomodulatory activities (5–7, 16). However, direct experimental validation is currently lacking. Consequently, the precise AS–PCa relationship remains undetermined.

Recent advances in bioinformatics provide powerful tools for elucidating complex environment–disease pathogenesis interactions (17, 18). Through multi-omics data integration and network-based analyses, these approaches have become key strategies for identifying molecular targets and pathways in disease mechanisms. Furthermore, machine learning algorithms show considerable potential for disease prediction and high-precision biomarker discovery. Therefore, this study uses an integrated approach combining multimodal bioinformatics, machine learning algorithms, and *in vitro* cellular assays to comprehensively investigate AS–PCa associations and elucidate underlying molecular mechanisms.

## Methods and materials

### Data sources

This study integrated transcriptomic profiles and matched clinical data from 1,098 PCa cases, including: (1) the TCGA-PRAD cohort (retrieved via UCSC Xena) and (2) three independent GEO cohorts (GSE21032, GSE70770, GSE116918). To enhance statistical power, we merged GSE21032 and GSE70770 into a combined GEO cohort, applying the ComBat algorithm (R sva package) to correct for technical batch effects. Patients lacking a biochemical recurrence (BCR) status or with <1-month follow-up were excluded to ensure data reliability. Comprehensive patient characteristics for all analytical cohorts are detailed in Supplementary Tables S1, S2.

### Identification of AS target genes

According to previous literature reports (4), seven ASs commonly used in China, the US, the EU, and other regions were selected: aspartame, acesulfame-K, sucralose, neohesperidin dihydrochalcone (NHDC), sodium cyclamate, neotame, and saccharin. These ASs served as search queries across three databases: Comparative Toxicogenomics Database (CTD), STITCH, and Super-PRED. Canonical SMILES strings and SDF structural formats for all ASs were retrieved from PubChem. Target prediction was performed using SwissTargetPrediction and Similarity Ensemble Approach (SEA) databases with SMILES data. Target prediction was performed using SwissTargetPrediction and Similarity Ensemble Approach (SEA) databases with SMILES data. SDF files were converted to mol2 format using Chem3D software, followed by target identification via PharmMapper and GalaxySagittarius platforms. Analyses focused on *Homo sapiens* targets meeting these thresholds: SwissTargetPrediction (probability >0.01), Super-PRED (probability ≥50%), and PharmMapper (Norm-Fit ≥0.7). Targets from all databases were integrated to establish unique target profiles for each AS. Targets from all seven ASs were aggregated into a unified target set for downstream analysis.

### Identification of prostate cancer targets

Transcriptomic data from TCGA included 534 PCa samples (483 tumors and 51 normal controls) (Supplementary Table S2). Differential expression analysis was performed using the DESeq2 R package with thresholds: |log2 fold change| > 0.65 and adjusted *p*-value <0.05. Significantly dysregulated genes were considered potential PCa therapeutic targets.

### AS–PCa target identification and PPI network construction

Overlapping targets between ASs and PCa were identified using Venn analysis. Protein–protein interactions (PPIs) among overlapping targets were analyzed using the STRING database (confidence score ≥0.4 thresholds), which integrates known and predicted protein associations, including physical interactions and functional linkages. Following PPI retrieval, non-essential targets were filtered to construct a target–protein interaction network. The interaction network (TSV format) was imported into Cytoscape 3.9.0 for visualization. Cytoscape’s Network Analyzer calculated topological properties (degree, closeness centrality, and betweenness centrality) to assess node importance. The “Analyze Network” module generated comprehensive topological datasets. Genes were ranked by degree scores, where higher values indicate greater network importance.

### Functional enrichment analysis

Gene Ontology (GO)^1^ categorizes gene functions into the cellular component (CC), molecular function (MF), and biological process (BP) domains. Kyoto Encyclopedia of Genes and Genomes (KEGG)^2^ systematically links genomic information with high-level functional pathways. GO and KEGG enrichment analyses were performed using the R package clusterProfiler.

### Developing AS–PCa prediction model using machine learning

To develop a high-accuracy diagnostic model: (1) TCGA-PCa cohort served as a training set; GSE and GSE116918 datasets as external validation sets; (2) Ten machine learning algorithms were implemented: random survival forest (RSF), elastic net (Enet), Lasso regression, ridge regression, stepwise Cox regression, CoxBoost, Cox partial least squares regression (plsRcox), SuperPC, generalized boosted regression models (GBMs), and survival support vector machine (survival-SVM); and (3) All 101 possible algorithm combinations were applied to AS–PCa genes. Models were ranked by mean C-index, with the highest-scoring model selected as optimal (19).

### Prediction model evaluation

Patients in TCGA-PRAD, GSE, and GSE116918 cohorts were stratified into low−/high-risk groups based on median risk scores. Survival differences were compared using Kaplan–Meier (K–M) curves. Predictive performance was validated across three independent datasets (TCGA, GSE, and GSE116918). Area under the curve (AUC) values were calculated to assess accuracy.

### Molecular docking

Molecular docking simulations elucidated interaction mechanisms between ASs and AS–PCa target proteins. AS molecular structures were retrieved from PubChem. Three-dimensional (3D) structures of AS–PCa target proteins were obtained from the AlphaFold Protein Structure Database. AutoDockTools 1.5.7 processed structures and performed docking simulations to predict binding modes, binding affinity (ΔG), and functional implications. Binding energies <0 kcal/mol indicate spontaneous binding, while energies <−5.0 kcal/mol indicate stable binding.

### Cell culture and treatment

Human normal prostate cells (RWPE-1) and PCa cells (DU145) were obtained from the Chinese Academy of Sciences Cell Bank. Cells were cultured as per the manufacturer’s protocol at 37°C in a humidified 5% CO₂ atmosphere. NHDC (Selleck, China) was dissolved in dimethyl sulfoxide (DMSO, Selleck) to prepare 100 mM stock solutions stored at 4°C. Stock solutions were diluted in a cell culture medium immediately before experiments to achieve a 30 μM final concentration for DU145 treatment. DMSO concentration was maintained below 0.1%, as concentrations ≤0.25% showed minimal cytotoxicity. Based on established cytotoxic profiles (20–23), we selected an NHDC concentration that demonstrates significant anti-tumor efficacy while maintaining minimal cytotoxicity in non-malignant cells.

### Western blotting

Following established protocols (24), total protein was isolated from RWPE-1 and DU145 cells using RIPA lysis buffer (Solarbio, China) supplemented with protease inhibitor cocktail (Yeasen, China). Proteins were resolved by 10% SDS-polyacrylamide gel electrophoresis and electrotransferred to PVDF membranes. Membranes were blocked with 5% non-fat dry milk for 1 h at room temperature, then incubated overnight at 4°C with an anti-MMP11 primary antibody (1:600; #30615-1-AP, Proteintech; Proteintech). After TBST washes, membranes were probed with species-matched horseradish peroxidase-conjugated secondary antibodies for 2 h at room temperature.

### EdU proliferation assay

The impact of NHDC on DU145 cell proliferation was assessed using the 5-ethynyl-2′-deoxyuridine (EdU) *In Vitro* Cell Proliferation Kit (Beyotime Biotechnology, Shanghai). Following 24-h incubation after seeding (3 × 10^3^ cells/well in 24-well plates), cells were pulse-labeled with 20 μL of EdU working solution for 2 h at 37°C. Cells were then fixed and stained according to the manufacturer’s protocol. EdU-positive cells were visualized using fluorescence microscopy, with representative images captured at 20 × magnification.

### Wound healing assay

NHDC-treated and untreated DU145 cells were cultured in complete medium (RPMI-1640 supplemented with 10% FBS) in 6-well plates until reaching 100% confluency. A uniform wound was created in the monolayer using a sterile 200 μL pipette tip. After PBS washing to remove dislodged cells, standardized wounds were established. Cells were maintained in low-serum migration medium (RPMI-1640 with 1% FBS) to minimize proliferation-related effects during wound closure assessment. Wound closure was monitored at 0 and 48 h post-wounding using phase-contrast microscopy (10 × objective).

### Transwell invasion assay

Consistent with our prior methodology (24), cells were suspended in serum-free medium and seeded into the upper chamber of Transwell® inserts (8-μm pore size). The lower chamber contained RPMI-1640 supplemented with 10% FBS as a chemoattractant. Following 24-h incubation at 37°C/5% CO₂, non-migratory cells on the upper membrane surface were removed by mechanical scraping with cotton swabs. Migrated cells on the lower surface were fixed with 4% paraformaldehyde and stained with 0.1% crystal violet for 20 min. Membranes were excised and mounted on slides, with migrated cells quantified by imaging five random fields per insert using an inverted microscope (10 × objective).

### Statistical analysis

All statistical analyses were performed using R software (version 4.2.2). Gene expression differences were assessed using both non-parametric Wilcoxon signed-rank tests and parametric paired Student’s t-tests, with statistical significance defined as a two-sided *p*-value of < 0.05. Survival analysis for disease-free survival (DFS) used Cox proportional hazards regression models and Kaplan–Meier curves with log-rank testing. To ensure reproducibility, all *in vitro* functional experiments included three biological replicates.

## Results

### Acquisition of AS–PCa targets

**Figure 1:**
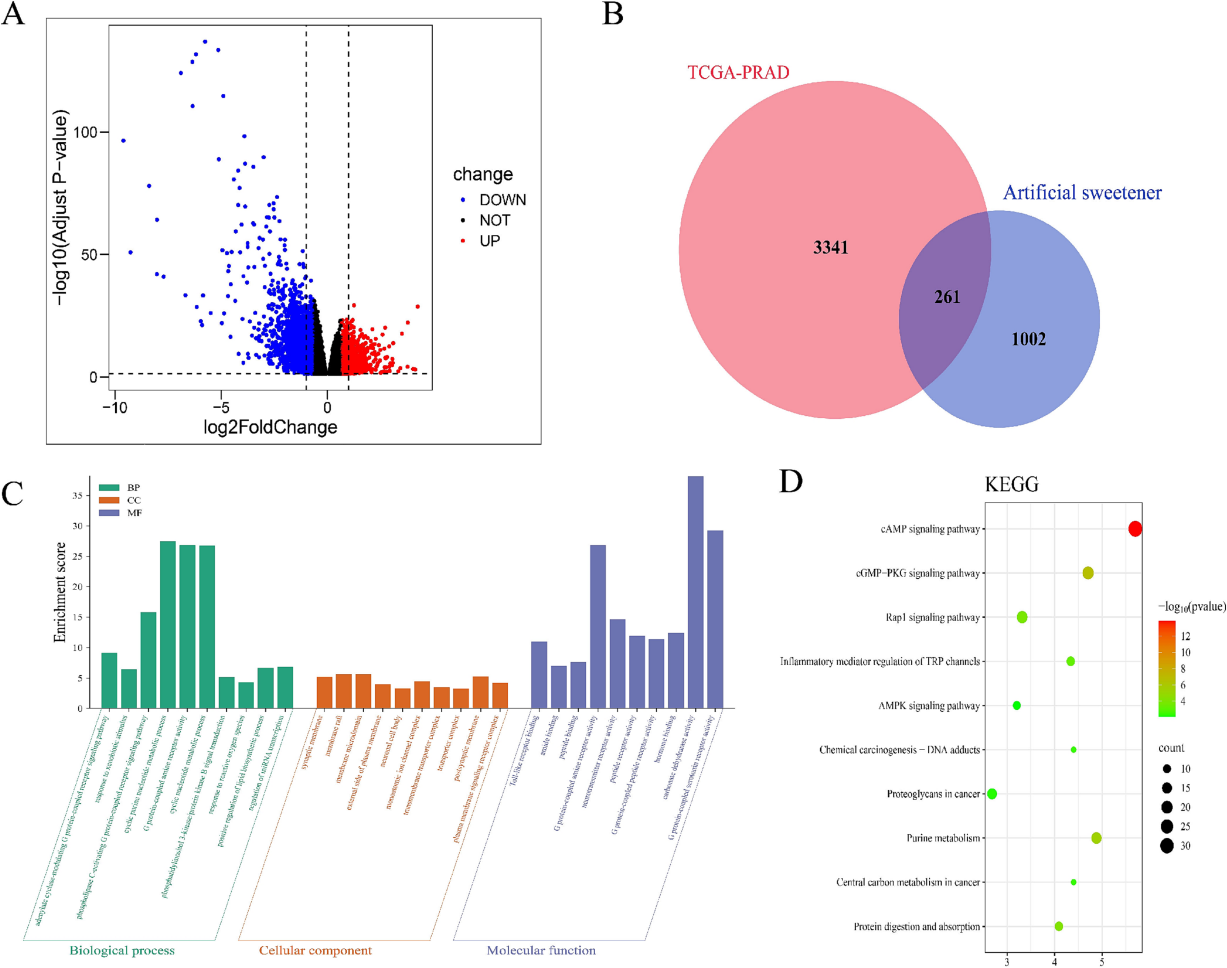
Identification and enrichment analysis of AS–PCa targets. **(A)** Volcanic map of differential gene expression in TCGA-PRAD, **(B)** AS–PCa common target Venn diagram, **(C)** GO enrichment analysis, and **(D)** KEGG enrichment analysis.

After integrating target prediction data from the above databases, we identified potential target genes for each artificial sweetener: aspartame, acesulfame-K, sucralose, NHDC, cyclamate, neotame, and saccharin. After duplicate removal, 1,263 unique AS-related target genes were retained. Subsequent differential expression analysis revealed 3,602 differentially expressed genes in tumor tissues (Supplementary Table S3, Figure 1A). Venn diagram analysis identified 261 overlapping AS–PCa genes (Figure 1B) representing potential therapeutic targets for artificial sweetener-mediated effects on prostate cancer.

### Enrichment analysis of AS–PCa targets

The GO enrichment analysis of 261 AS–PCa genes revealed important roles in biological processes, including the cGMP signaling pathway, cell proliferation, hypoxia response, microRNAs in cancer, and biosynthetic pathways (Figure 1C). The KEGG pathway analysis further identified the cAMP signaling pathway, cGMP-PKG signaling pathway, Rap1 signaling pathway, TRP channels involved in inflammatory mediator regulation, AMPK signaling pathway, chemical carcinogenesis-DNA adducts, proteoglycans in cancer, purine metabolism, central carbon metabolism in cancer, protein digestion, and absorption (Figure 1D). These findings suggest that artificial sweeteners may affect PCa by modulating inflammatory, apoptotic, and oncogenic pathways.

### Construction of PPI network for AS–PCa targets

**Figure 2:**
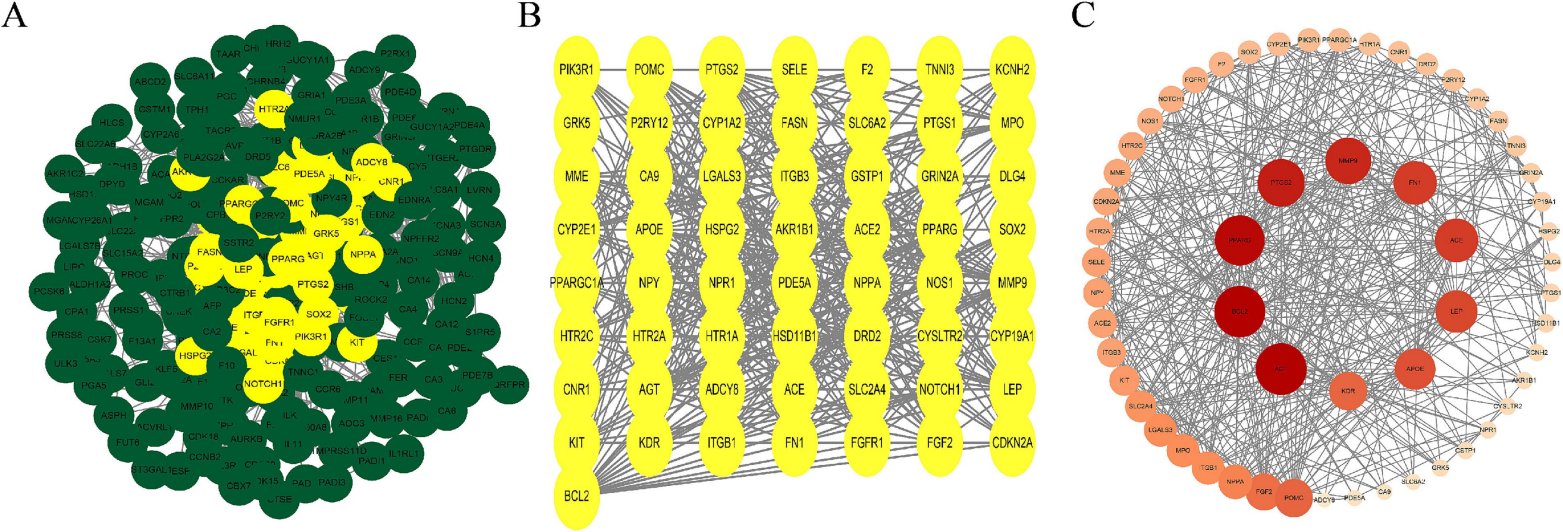
AS–PCa protein mapping and network core protein screening. **(A)** Protein-protein interaction (PPI) network of all common targets, with hub targets highlighted in yellow; **(B)** PPI network of the hub targets; **(C)** PPI network of hub targets ranked by Degree, with deeper colors and larger nodes indicating higher Degree scores.

We imported 261 overlapping AS–PCa target genes into the STRING database for PPI analysis, applying a confidence threshold of ≥ 0.4. A total of 257 AS–PCa targets remained after filtering disconnected nodes. The PPI network was visualized using Cytoscape 3.10.3. Nodes were arranged by degree, with darker colors and larger diameters indicating stronger protein interactions. Network analysis identified 10 core targets in the AS–PCa interaction network: AGT, BCL2, PPARG, PTGS2, MMP9, FN1, ACE, LEP, APOE, and KDR (Figures 2A–C). This visualization provided a clear overview of the interactions between the key targets and provided valuable insights for further investigation of the molecular mechanisms linking ASs and PCa.

### Establishment and evaluation of the AS–PCa prediction model

**Figure 3:**
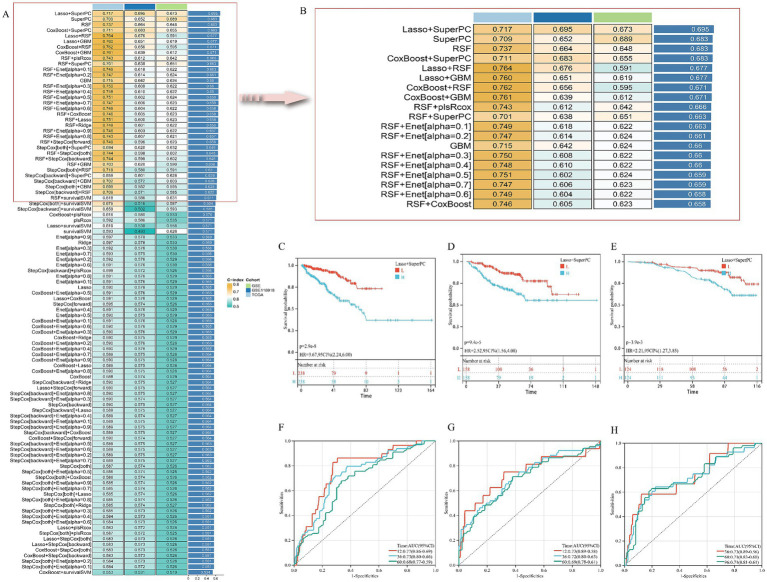
Establishment and validation of risk prediction model related to AS–PCa target. **(A,B)** Average C-index of 101 robotic algorithm combinations; **(C,F)** TCGA-PRAD cohort; **(D,G)** GSE cohort; **(E,H)** GSE116918 cohort.

Univariate Cox regression initially identified 52 AS–PCa genes associated with disease-free survival (DFS) in the TCGA-PRAD cohort (Supplementary Table S4). Systematic evaluation of 101 algorithm combinations identified the Lasso-SuperPC hybrid model as optimal, incorporating 18 key genes (Figures 3A,B, Supplementary Figure S1A) with a mean C-index of 0.695. Kaplan–Meier analysis demonstrated significantly shorter DFS in high-risk groups (stratified by median risk score) across TCGA-PRAD (Figure 3C), GSE (Figure 3D), and GSE116918 (Figure 3E) cohorts. Time-dependent ROC curves confirmed robust predictive performance for DFS: TCGA-PRAD (AUC: 1-year 0.77, 3-year 0.73, and 5-year 0.68; Figure 3F), GSE (1-year 0.73, 3-year 0.72, 5-year 0.69; Figure 3G), and GSE116918 (3-year 0.73, 5-year 0.71, 8-year 0.71; Figure 3H).

### Survival analysis of core targets in the AS–PCa prediction model

**Figure 4:**
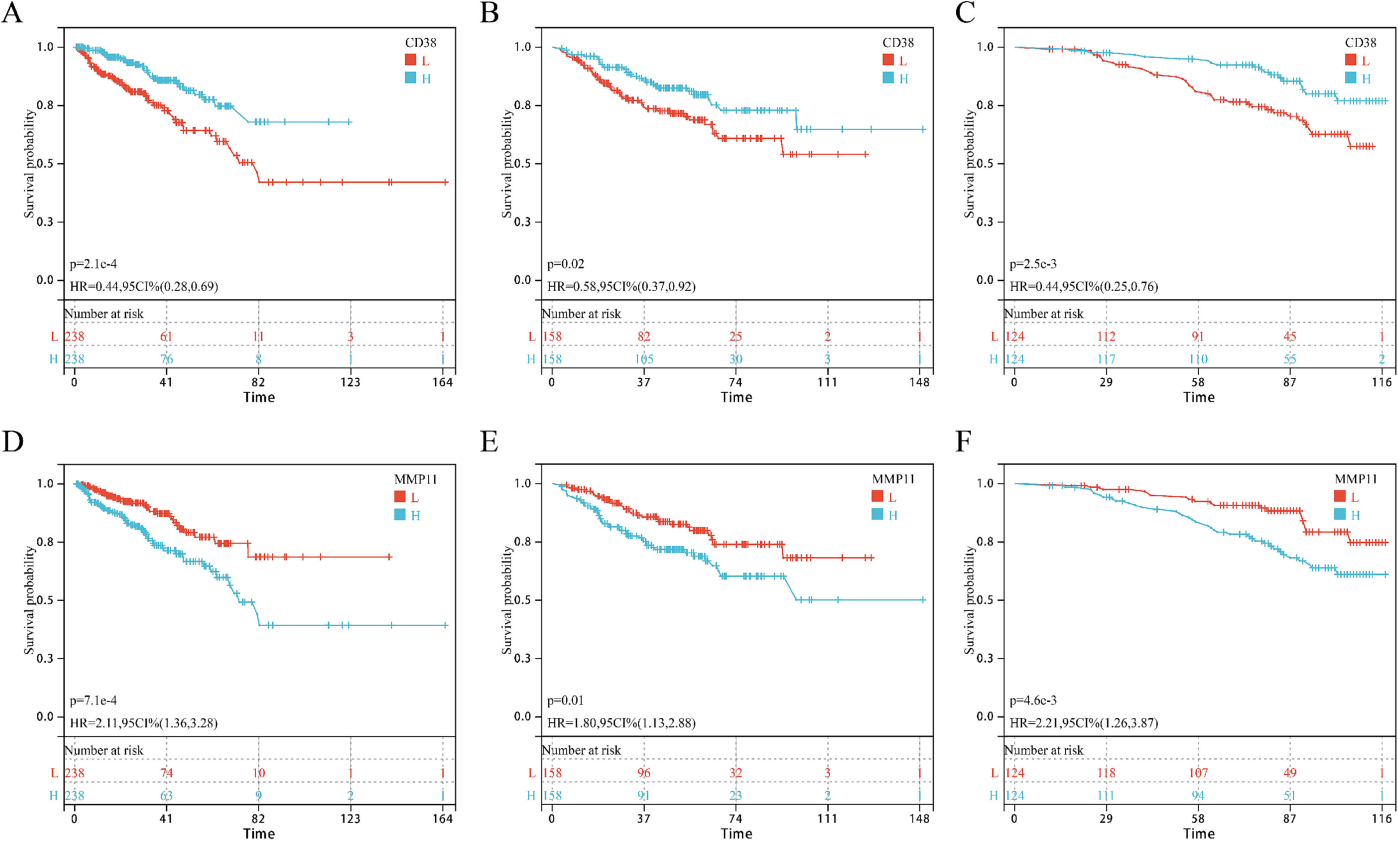
KM survival curves of core genes CD38 and MMP11 in three cohorts. **(A–C)** CD38; **(D–F)** MMP11; **(A,D)** TCGA-PRAD cohort; **(B,E)** GSE cohort; **(C,F)** GSE116918 cohort.

Univariate Cox regression in GSE and GSE116918 cohorts identified five consensus prognostic genes (Supplementary Figure S1) consistently associated with DFS across all cohorts: CCNB2, CD38, CDC20, MMP11, and PDE4D. Kaplan–Meier analysis revealed that only CD38 (Figures 4A–C) and MMP11 (Figures 4D–F) showed significantly shorter DFS with high expression in all three cohorts. Building upon our prior validation of MMP11’s critical pro-tumorigenic role (24), we selected MMP11 as the principal investigative focus for subsequent mechanistic exploration.

### The influence of seven types of ASs on the core targets

**Figure 5:**
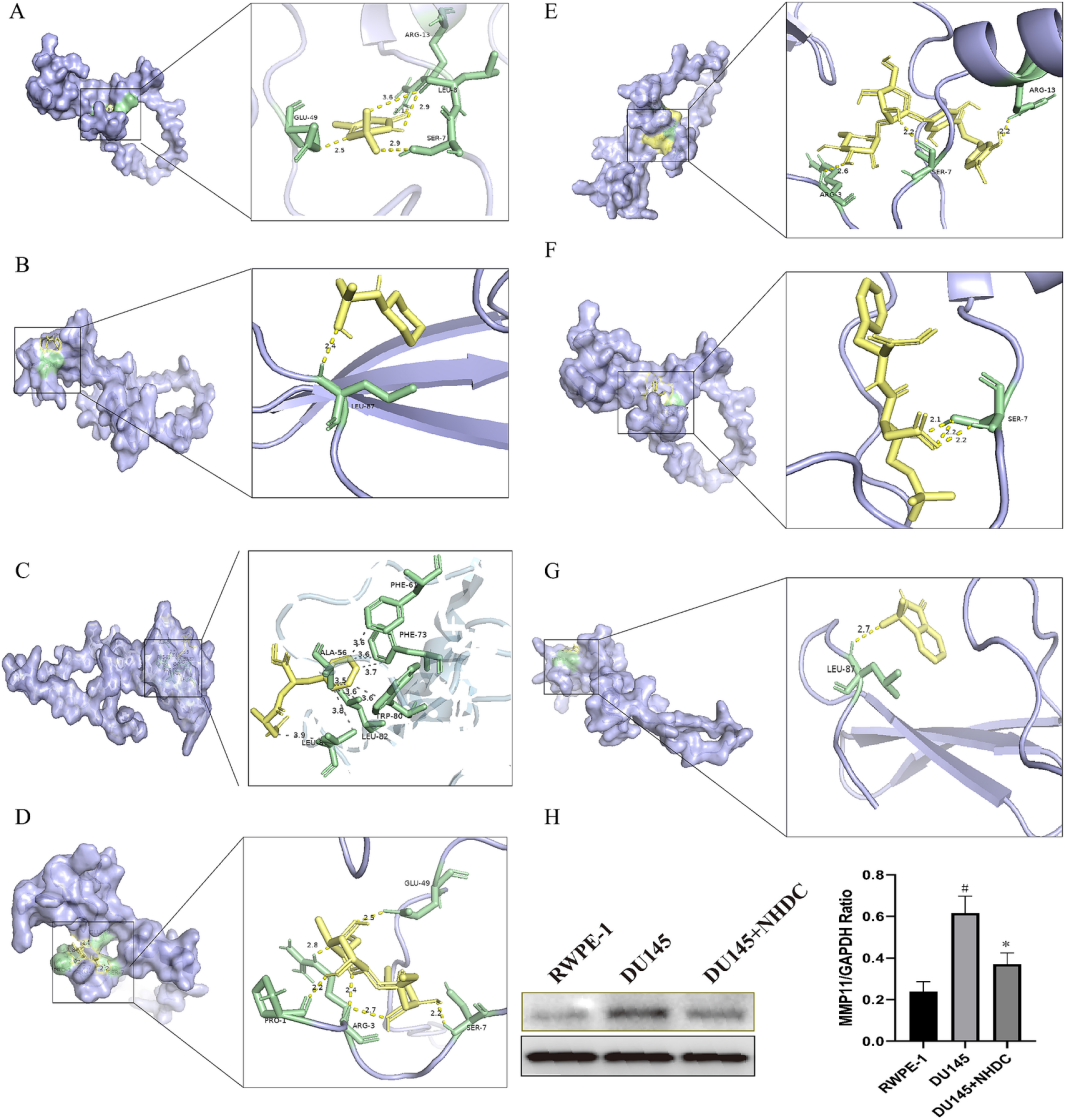
Impact of seven types of AS on the core protein MMP11. **(A)** acesulfame-K; **(B)** cyclamate; **(C)** aspartame; **(D)** sucralose; **(E)** NHDC; **(F)** neotame; **(G)** saccharin; **(H)** The effect of NHDC on the expression of MMP11.

Molecular docking assessed binding interactions between seven AS compounds and MMP11. All seven ASs showed spontaneous binding (ΔG < 0 kcal/mol), with five exhibiting stable binding (ΔG < −5 kcal/mol): acesulfame-K (−4.2; Figure 5A), sodium cyclamate (−4.5; Figure 5B), aspartame (−5.0; Figure 5C), sucralose (−5.2; Figure 5D), NHDC (−7.1; Figure 5E), neotame (−5.7; Figure 5F), and saccharin (−5.6; Figure 5G). These results indicate that direct AS–MMP11 interactions may modulate PCa-related biological processes. Due to its highest binding stability, NHDC was selected for functional validation.

### The effect of NHDC on PCa *in vitro*

**Figure 6:**
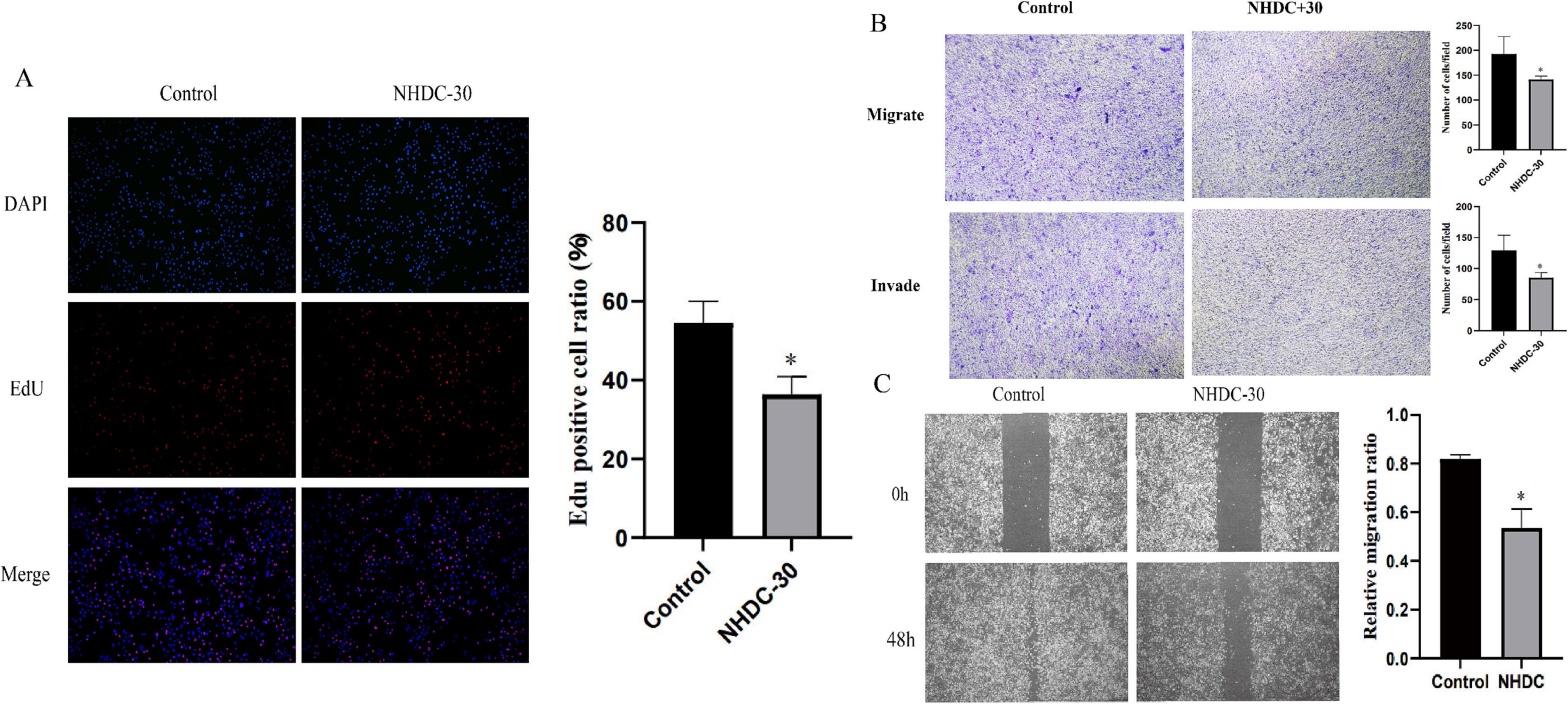
Effect of NHDC on the biological behavior of DU145 cells *in vitro*. **(A)** EdU assay; **(B)** Transwell assay; **(C)** Wound healing assay.

WB analysis showed that protein levels of MMP11 in PCa cells were reduced after NHDC treatment (Figure 5H). Functional assays showed that NHDC intervention significantly inhibited proliferation (EdU assay) (Figure 6A), invasion (Transwell assay) (Figure 6B), and migration (Scratch Healing assay) (Figure 6C) of DU145 cells. These findings support the tumor suppressor effect of NHDC in PCa.

## Discussion

ASs are extensively incorporated into foods and beverages due to their low-calorie properties and high-intensity sweetness. The global AS market is projected to approach $10 billion by 2028 (1–3). Widespread AS usage has raised concerns about environmental contamination and human health impacts, particularly carcinogenic risks (8–10). Several studies suggest potential carcinogenic hazards from AS consumption (8–10, 25, 26). However, inconsistent epidemiological validation in human populations leaves the carcinogenic significance of ASs scientifically contentious. Given large-scale production and extensive use, universal human exposure to potential AS-related hazards is increasingly unavoidable. Thus, elucidating AS health risks remains crucial for developing effective risk-mitigation strategies.

PCa ranks among the leading malignancies in men worldwide by both incidence and mortality (11–13). Its pathogenesis involves complex pathophysiological mechanisms, with oxidative stress, chronic inflammation, and epigenetic modifications being widely recognized as pivotal factors (13, 14, 27, 28). Interestingly, prior research highlights that certain ASs exhibit pharmacological properties including antipyretic, analgesic, anti-inflammatory, and immunomodulatory effects (5–7), which may beneficially suppress PCa initiation and progression (13, 14, 27, 28), though direct laboratory evidence remains lacking. In this study, we used a multidisciplinary approach integrating bioinformatics, machine learning, molecular docking, and *in vitro* cellular assays to reveal potential AS–PCa connections. We further identified key genes and their interaction networks, providing novel insights into AS roles in PCa.

Our analysis identified 261 AS–PCa bridging genes. KEGG and GO enrichment analyses suggest that ASs may influence PCa through modulation of inflammatory responses, cell proliferation, biosynthetic pathways, hypoxia responses, and cGAS-STING signaling. Subsequently, machine learning algorithms developed a robust risk model using 52 PRAD progression-associated genes, demonstrating strong predictive performance for DFS. Finally, our research further identified CD38 and MMP11 as reliable survival biomarkers.

MMP11 (stromelysin-3), a matrix metalloproteinase family member, critically regulates extracellular matrix degradation and remodeling (29, 30). Elevated MMP11 expression correlates with poor outcomes across multiple cancers (31–33). We observed a similar trend: MMP11 overexpression in PCa correlates with shorter progression-free survival. Previous studies confirmed that suppressing MMP11 expression effectively inhibits PCa cell proliferation, migration, and invasion, underscoring its crucial role in PCa pathogenesis (34–36). Intriguingly, molecular docking revealed spontaneous binding between all seven AS compounds and MMP11, with NHDC showing the highest binding stability. Western blotting confirmed significantly reduced MMP11 protein levels in NHDC-treated PCa cells, providing experimental evidence that ASs may exert anti-PCa effects via MMP11 modulation.

NHDC, a widely used, safe, low-calorie, non-nutritive artificial flavor derived from neohesperidin dihydrochalcone (37), demonstrates anti-inflammatory effects by reducing LPS-induced cytokine production, modulating mitochondrial oxidative phosphorylation, and exhibiting free-radical scavenging and ROS-inhibitory activities (38–40). These pharmacological properties position NHDC as an antitumor drug candidate. Indeed, Kim et al. demonstrated that NHDC inhibits proliferation and invasion in triple-negative breast cancer cells (23). Our study further validates these findings, showing that NHDC similarly suppresses PCa cell proliferation, migration, and invasion while inhibiting the expression of the tumor progression factor MMP11. To our knowledge, this represents the first investigation of NHDC’s effects on prostate cancer pathogenesis, highlighting its novel implications for PCa management.

Although MMP11 remains our primary focus based on previous studies, the critical role of CD38 in prostate cancer development warrants concurrent investigation. As a multifunctional extracellular enzyme, CD38 regulates cellular NAD homeostasis through NAD catabolism (41, 42). CD38 dysfunction is associated with various pathophysiological processes, including aging, metabolic disorders (such as obesity/diabetes), cardiovascular disease, and chronic inflammation (41, 42). Its tumor-regulatory function in prostate cancer has been confirmed (43–45). First, recent laboratory evidence suggests that the epigenetic silencing of CD38 in PCa cells enhances NAD availability, thereby conferring a survival advantage to tumor-initiating cells by improving mitochondrial function (43, 44). Similar previous studies have emphasized that CD38 expressed by immune cells in the microenvironment can promote immune suppression through adenosine production, thereby facilitating PCa progression (45). Notably, no studies have investigated the regulatory effects of artificial sweeteners (especially natural derivatives such as NHDC) on CD38. Given the importance of CD38, further clarification of the specific regulatory effects of artificial sweeteners (especially natural derivatives such as NHDC) on it may be beneficial for new drug development in PCa. Therefore, future studies should consider: (1) validating the responsiveness of CD38 to sweeteners and (2) elucidating the mechanisms of AS-CD38 crosstalk.

This study offers significant methodological advances over prior research (4, 9, 15): First, our integrated approach combining bioinformatics, machine learning, molecular docking, and *in vitro* validation substantially enhances scientific rigor. Earlier studies predominantly relied on database-derived epidemiological surveys focusing on single synthetic artificial sweeteners. However, such approaches face inherent limitations in prostate cancer research, where pathogenesis involves complex gene–environment interactions. Dietary confounders remain challenging to control in observational studies, potentially compromising the accuracy of epidemiological conclusions. Second, even the recent bioinformatic analysis of seven sweeteners in PCa (4) by Xie et al. used limited methodologies: core target screening lacked machine learning precision, and computational predictions remained experimentally unvalidated. Crucially, our study provides the first experimental evidence of NHDC’s anti-tumor effects against PCa cells *in vitro*, potentially mediated through MMP11 suppression. These findings offer translational implications for dietary management (e.g., recommending natural-origin sweeteners such as NHDC) and novel therapeutic development for prostate cancer patients.

We acknowledge several limitations: First, target identification relied solely on database predictions susceptible to algorithmic bias, potentially compromising artificial sweetener (AS) target accuracy. Second, experimental validation was restricted to naturally derived NHDC, limiting generalizability to synthetic AS such as aspartame. Third, *in vitro* models cannot replicate *in vivo* absorption, distribution, metabolism, and excretion (ADME) processes, particularly relevant given evidence of carcinogenic AS metabolites. Fourth, while NHDC concentrations followed literature precedents, biological heterogeneity across cancer lineages may limit dose optimization. Future studies should incorporate detailed dose–response and toxicity profiling. Fifth, although Western blotting confirmed MMP11 suppression, upstream regulatory mechanisms (transcriptional regulation and proteasomal degradation) remain unexplored. Finally, while molecular docking predictions aligned with WB results, we cannot exclude indirect effects through intermediate molecules. Direct binding validation (isothermal titration calorimetry [ITC] or surface plasmon resonance [SPR]) remains essential to confirm MMP11’s mechanistic centrality.

## Conclusion

This study comprehensively investigated the relationship between ASs and PCa using an integrated approach that combined bioinformatics, machine learning, molecular docking, and *in vitro* cellular assays. Our findings demonstrate that ASs influence PCa initiation and progression through multiple molecular targets and signaling pathways. We discovered that NHDC exhibits tumor-suppressive effects against PCa for the first time, identifying MMP11 as a key mechanistic mediator. Furthermore, we developed an 18-gene risk prediction model based on core AS–PCa interactions, offering promising targets for PCa early detection, prognostic assessment, and targeted therapy. Collectively, this study provides novel insights into the AS–PCa relationship. Future research should use comprehensive *in vitro* and *in vivo* models to validate AS effects on PCa pathogenesis and elucidate precise molecular mechanisms underlying AS-mediated MMP11 regulation.

## Data Availability

The original contributions presented in the study are included in the article/Supplementary material, further inquiries can be directed to the corresponding author.

## Footnotes

^1^ http://geneontology.org

^2^ https://www.kegg.jp

## Supplementary material

SUPPLEMENTARY FIGURE S1 Screening of core genes related to DFS progression in all three cohorts. **(A)** 18 key genes identified through the combination of Lasso+SuperPC; **(B)** Common prognostic gene.
